# Organ retrieval and collection of health information for donation: The ORCHID dataset

**DOI:** 10.1038/s41597-025-06435-1

**Published:** 2026-01-13

**Authors:** Hammaad Adam, Tom Pollard, Vinith Suriyakumar, Benjamin Moody, Jan Niklas Adams, Jennifer Erickson, Greg Segal, Matthew Wadsworth, Ashia Wilson, Marzyeh Ghassemi

**Affiliations:** 1 Massachusetts Institute of Technology, Cambridge, 02139 MA USA; 2 RWTH Aachen University, Aachen, Germany; 3 Federation of American Scientists, Washington, 20036 DC USA; 4 Organize, White Plains, 10604 NY USA; 5 Life Connection of Ohio, Maumee, 43537 OH USA

**Keywords:** Health services, Outcomes research

## Abstract

Organ transplantation is a life-saving procedure for patients with advanced diseases. However, the demand for transplants far exceeds the supply of donated organs, and there are currently over 100,000 people waiting for a transplant in the United States. The lives of these patients depend on the efficacy of organ procurement organizations (OPOs), which coordinate the recovery of organs from deceased donors. However, many studies have found high variation in performance amongst OPOs. Coordinating data collection and analysis across OPOs is a crucial first step in closing performance gaps and achieving more effective organ donation. In 2021, the Federation of American Scientists announced a collaboration in which six OPOs committed to an unprecedented level of data sharing. This paper marks the release of ORCHID, this collaboration’s first public dataset. ORCHID comprises detailed information on referrals for donation, procurement outcomes, and process data from the participating OPOs. Our goal in releasing this data is to promote research that leads to better services for organ donors, donor families, and patients waiting for transplants.

## Background

Organ transplantation is an extraordinary achievement of modern medicine, offering life to patients in organ failure. This success has only been possible due to the generosity of hundreds of thousands of organ donors. In the United States, the responsibility of recovering organs for transplantation from deceased donors lies with fifty-six organ procurement organizations (OPOs), each mandated by federal law to carry out organ recovery in a designated service area. For every successful donation, an OPO facilitates authorization, recovery of donor organs, and delivery to the transplant hospital (https://unos.org/transplant/opos-increasing-organ-donation/).

Perhaps the most significant challenge in transplantation today lies in the disparity between demand for organs and their scarce availability, regardless of organ type^1^. In 2021 alone, more than 11,000 wait-listed patients—more than 30 every day—were removed from the transplant waiting list either because they died or became too unwell to undergo transplantation, according to Organ Procurement and Transplantation Network (OPTN) data as of November 6, 2023. Over 103,000 adults and children are on the national transplant waiting list today, the majority of whom are unlikely to receive a transplant within a year^1^. Even more concerningly, a large proportion of patients waiting for a transplant are from underserved populations; for example, over 60% of currently waitlisted patients are Black, Indigenous, or People of Color (BIPOC)^1^.

Despite the growing need for donor organs, the current system of organ procurement has previously been documented to be inefficient and variable^2–6^. High variability in OPO performance has been attributed to a multitude of factors including differences in organizational structures and operational approaches and a lack of oversight and accountability. These inefficiencies contribute to an estimated 28,000 transplant-viable organs going untransplanted every year^7^.

Current research in organ procurement has been severely limited by a lack of publicly available data. Coordinating data collection and analysis is thus a crucial first step to improving performance. In 2021, the Federation of American Scientists (FAS) announced a collaboration in which six OPOs agreed to an unprecedented level of data sharing^8^. These OPOs underscored their commitment to principles of transparency, accountability, and equity by committing to a public release of information including referrals for donations, referral outcomes, and procurement and organ recovery data from organ recovery centers. In this paper we introduce the Organ Retrieval and Collection of Health Information for Donation (ORCHID) dataset, a public, granular multi-center dataset on organ procurement.

## Methods

The Organ Retrieval and Collection of Health Information for Donation (ORCHID) dataset is the result of a collaboration between six OPOs, researchers at Massachusetts Institute of Technology (MIT), Organize, and the Federation of American Scientists. ORCHID contains case-level performance data routinely collected during procurement between January 2015 and December 2021, creating new opportunities for collaborative analysis of the organ procurement process.

In this section, we outline the processes that create OPO data, our specific methods for acquiring data from six collaborating OPOs, the de-identification process used, data harmonization efforts to create a meaningful dataset for release, and the details of how to gain access to ORCHID through the PhysioNet website.

### Organ Procurement Processes

**Fig. 1:**
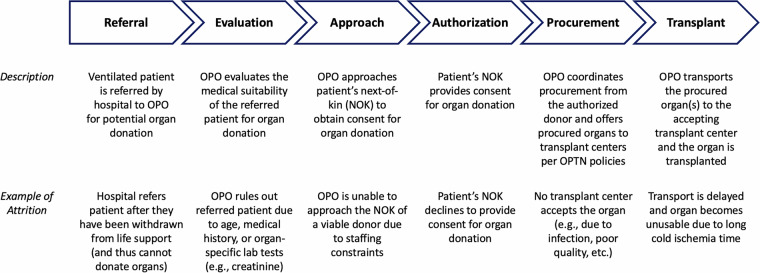
Stages of the organ donation process overseen by organ procurement organizations. Broadly, the process can be viewed as having six distinct phases: Referral; Evaluation; Approach; Authorization; Procurement; and Transplant. NOK is Next of Kin; OPO is Organ Procurement Organization; OPTN is Organ Procurement and Transplantation Network.

The organ procurement process conducted by OPOs can be broadly grouped into six stages (Fig. 1), outlined below:

- **Referral**: When a ventilated patient in a hospital meets certain clinical triggers, they are referred to a designated OPO. These clinical triggers are agreed upon between the OPO and the hospital (e.g., patient has Glasgow Coma Score ≤5).
- **Evaluation**: The OPO evaluates the referral and makes an initial assessment on the patient’s suitability for organ donation.
- **Approach**: A representative of the OPO approaches the patient’s next-of-kin to obtain consent for donation. If the patient had provided consent during their lifetime (e.g., by entering in a donor registry), the OPO communicates this directive to the next-of-kin.
- **Authorization**: The OPO formally documents authorization obtained via the donor registry or from the patient’s next-of-kin.
- **Procurement**: The OPO optimizes organ viability and coordinates the procurement of organs from the deceased donor patient. The OPO offers the organ(s) to patients on the waiting list per the policies of the Organ Procurement and Transplantation Network (OPTN).
- **Transplant**: The OPO or transplant center arranges transport to the accepting transplant center and transplantation proceeds.

During the course of their work, OPOs record detailed information on factors such as patient demographics, timing of key events, and the outcomes of procurement. These data are recorded in relational databases that underpin two donor information systems: the iTransplant platform (used by 5 of 6 of the participating OPOs, https://www.invitahealth.com/solutions/donation-transplant-management/transplant/) and the TrueNorth platform (used by the remaining OPO, https://www.lifelogics.org/products).

### Data Acquisition From OPOs

Prior to acquiring data for the project, we sought Institutional Review Board approval for the transfer, storage, analysis, and release of organ procurement data from six OPOs: Southwest Transplant Alliance, Mid-America Transplant, OurLegacy Organ and Tissue Donation Services, Louisiana Organ Procurement Agency, Life Connection of Ohio, and Donor Network West. The project received approval by the Massachusetts Institute of Technology Committee on the Use of Humans as Experimental Subjects (IRB protocol: 2201000540A001). Because the data were collected retrospectively from deceased organ donor referrals and subsequently de-identified, informed consent from individual patients or families was not required.

Guidelines for extracting data from the iTransplant and TrueNorth database systems were developed by the project team. These guidelines were sent to data managers at each of the participating OPOs, and a call was scheduled to coordinate the transfer process as needed. Each OPO was asked to first extract their Microsoft SQLServer database to a.bacpac file, which was securely transferred to servers at MIT that were approved for holding protected health information. Data files were loaded into a Microsoft SQLServer relational database system, and a subset of relevant data were extracted from the database using SQL queries run in Python (version 3.6.8) using the pyodbc package. The data was then aggregated in Python into a harmonized relational database structure to get to the final data files across all six OPOs.

### De-identification

All data were de-identified in accordance with Health Insurance Portability and Accountability Act (HIPAA) using structured data cleansing and date shifting^9^. This process required the removal of all eighteen of the identifying data elements listed in HIPAA, including patient name, address, and dates.

Patients over the age of 89 had their exact age obscured. Dates were shifted into the future by a random offset for each individual patient. The date-shifting method preserves intervals for each patient (for example, the true time interval between OPO referral and approach can be computed by subtracting the referral timestamp from the approach timestamp) and time of day. Note that to allow researchers to track annual trends, we provide the real (i.e., not time-shifted) year of referral as a separate field, since this is not a HIPAA identifier.

### Multi-site Data Harmonization

The information captured across the six OPOs was similar, allowing relatively straightforward harmonization of the structure and terminologies. The relevant column names in each OPO’s database were identified through discussions with OPO staff. One major difference between OPOs was the documentation of whether the patient’s next-of-kin was approached. Only two OPOs had an explicit binary field that indicated approach. We worked with staff at the other four OPOs to identify proxies to impute this variable. We determined that the accepted use of the database configuration was to consider a patient’s next-of-kin to have been approached if: (a) they had a non-null value for a field that contained the date-time of the approach, or (b) an approach outcome (i.e., a response of “Yes” or “No” to: “Authorization given?”). This definition provided a proxy for approach that aligned well with OPO practice.

### External Data Sources

The OPO databases only contain data on patients who were referred to the OPOs. However, not all donation-eligible deaths are referred to OPOs, and the number of deaths that go unreferred varies widely both between OPOs and within OPOs by year and geography^4,10^. The total number of referrals received by an OPO is thus not a consistent denominator on which to judge OPO performance.

To remedy this shortcoming, we use external data sources to provide annual estimates of the number of deaths in an OPO’s designated service area (DSA) that were consistent with organ donation, as per the Cause, Age, and Location Consistent (CALC) definition provided by the Centers for Medicare & Medicaid Services (CMS)^11^. These estimates were derived from county-level death statistics obtained from the Centers for Disease Control and Prevention (CDC) WONDER tool. CMS uses the restricted-access National Center for Health Statistics (NCHS) detailed Multiple Cause of Death (MCOD) files to calculate CALC deaths for each OPO. ORCHID uses the CDC Wonder Tool which closely aligns with the MCOD data, though the datasets are not identical. Note that CALC deaths likely overestimate true donation potential; internal OPO estimates suggest that only 30-40% of the CALC deaths in a DSA are viable candidates for donation. However, CALC still represents a consistent denominator on which to assess OPO performance as it relies on public data, not internal OPO metrics^12,13^. CMS will use CALC deaths to assess OPO performance under the new OPO Final Rule^11^; ORCHID thus allows researchers to measure performance in a way that is consistent with federal regulation.

We make three important notes on our CALC death estimates. First, the CDC WONDER tool censors the exact number of deaths in counties that have fewer than 10 deaths. We thus provide a lower bound on CALC deaths in an OPO’s DSA by assuming that each of these counties had exactly 1 death, an upper bound by assuming that each of these counties had exactly 9 deaths, and a median estimate by assuming that each of these counties had exactly 4 deaths. Second, a small number of counties were shared by two OPOs. For these counties, we divided the county-level deaths between the relevant OPOs using the split specified by CMS in their annual OPO reports^14^. Third, WONDER does not capture deaths of nonresidents (e.g. nonresident aliens, nationals living abroad), and so may underestimate CALC deaths in some counties.

## Data Records

ORCHID v2.1.1 has been made publicly available on PhysioNet, a popular repository of clinical and physiological data maintained by researchers at the Laboratory for Computational Physiology in Massachusetts Institute of Technology^15^.

### Patient Characteristics

ORCHID contains data associated with 133,101 distinct organ donation referrals between 2015 and 2021. The median age of patients is 61 years. 59% percent of patients are male and 60% patients are white. The most common cause of death is recorded as anoxia (30%). Table 1 provides detailed information on the demographics of referred patients, stratified by OPO.

**Table 1:** Demographics of potential donors in the ORCHID dataset.

	OPO1	OPO2	OPO3	OPO4	OPO5	OPO6	Overall
	(N = 32,148)	(N = 16,144)	(N = 12,516)	(N = 33,641)	(N = 15,738)	(N = 22,914)	(N = 133,101)
**Age**							
Under 25	2,611 (8.1%)	883 (5.5%)	725 (5.8%)	2,108 (6.3%)	1,218 (7.7%)	1,169 (5.1%)	8,714 (6.5%)
25-44	4,537 (14.1%)	2,331 (14.4%)	1,903 (15.2%)	4,826 (14.3%)	2,678 (17.0%)	2,990 (13.0%)	19,265 (14.5%)
45-64	12,310 (38.3%)	6,285 (38.9%)	4,916 (39.3%)	12,636 (37.6%)	6,285 (39.9%)	8,277 (36.1%)	50,709 (38.1%)
65-75	7,825 (24.3%)	4,423 (27.4%)	3,674 (29.4%)	8,227 (24.5%)	4,141 (26.3%)	6,958 (30.4%)	35,248 (26.5%)
Over 75	4,815 (15.0%)	2,221 (13.8%)	1,286 (10.3%)	5,837 (17.4%)	1,406 (8.9%)	3,516 (15.3%)	19,081 (14.3%)
Missing	50 (0.2%)	1 (0.0%)	12 (0.1%)	7 (0.0%)	10 (0.1%)	4 (0.0%)	84 (0.1%)
**Gender**							
Female	13,355 (41.5%)	6,982 (43.2%)	5,325 (42.5%)	13,159 (39.1%)	6,514 (41.4%)	9,434 (41.2%)	54,769 (41.1%)
Male	18,767 (58.4%)	9,161 (56.7%)	7,185 (57.4%)	20,462 (60.8%)	9,221 (58.6%)	13,475 (58.8%)	78,271 (58.8%)
Unknown	26 (0.1%)	1 (0.0%)	6 (0.0%)	20 (0.1%)	3 (0.0%)	5 (0.0%)	61 (0.0%)
**Race**							
White	16,316 (50.8%)	8,635 (53.5%)	9,856 (78.7%)	16,840 (50.1%)	12,212 (77.6%)	15,829 (69.1%)	79,688 (59.9%)
Black	6,485 (20.2%)	6,956 (43.1%)	2,204 (17.6%)	3,015 (9.0%)	2,941 (18.7%)	3,579 (15.6%)	25,180 (18.9%)
Hispanic	8,360 (26.0%)	357 (2.2%)	318 (2.5%)	8,493 (25.2%)	199 (1.3%)	3,001 (13.1%)	20,728 (15.6%)
Other/Unknown	987 (3.1%)	196 (1.2%)	138 (1.1%)	5,293 (15.7%)	386 (2.5%)	505 (2.2%)	7,505 (5.6%)
**Death Type**							
Brain death	3,152 (9.8%)	1,202 (7.4%)	778 (6.2%)	3,241 (9.6%)	1,964 (12.5%)	1,594 (7.0%)	11,931 (9.0%)
Cardiac death	28,996 (90.2%)	14,942 (92.6%)	11,738 (93.8%)	30,400 (90.4%)	13,774 (87.5%)	21,320 (93.0%)	121,170 (91.0%)
**Cause of Death**							
Anoxia	8,400 (26.1%)	4,286 (26.5%)	6,519 (52.1%)	10,569 (31.4%)	2,568 (16.3%)	8,022 (35.0%)	40,364 (30.3%)
Cerebrovascular accident/Stroke	4,936 (15.4%)	1,606 (9.9%)	1,738 (13.9%)	6,094 (18.1%)	0 (0%)	3,209 (14.0%)	17,583 (13.2%)
Head trauma	2,806 (8.7%)	880 (5.5%)	1,200 (9.6%)	3,171 (9.4%)	969 (6.2%)	1,794 (7.8%)	10,820 (8.1%)
Other	3,826 (11.9%)	6,623 (41.0%)	2,981 (23.8%)	8,951 (26.6%)	2,268 (14.4%)	9,867 (43.1%)	34,516 (25.9%)
Unknown	12,180 (37.9%)	2,749 (17.0%)	78 (0.6%)	4,856 (14.4%)	9,933 (63.1%)	22 (0.1%)	29,818 (22.4%)

OPO1 to OPO6 are inaugural members of the Federation of American Scientists (FAS) Organ Procurement Organization (OPO) Innovation Cohort. Each OPO covers a different service area in the United States. Created with TableOne^20^.

### Data Structure

ORCHID is a relational database consisting of nine tables, each of which is provided as a comma separated value (CSV) file. Data falls into three broad categories: “OPO Referrals”, “OPO Events”, and “OPO Deaths”, which are outlined below.

- **OPO Referrals** (OPOReferrals.csv): documents each patient referred to an OPO and records demographics, cause-of-death, and procurement outcomes.
- **OPO Events** (<Chart_type>Events.csv): describes events charted by OPO staff during the procurement process, including laboratory tests, vital signs, and clinical notes.
- **OPO Deaths** (OPODeaths.csv): reports a count of all CALC deaths in each OPO’s service area, whether or not they were referred to the OPO for donation.

Each patient is assigned a newly generated PatientID that does not correspond to any identifiers in the source OPO databases. This identifier was created solely for the ORCHID release to enable linking across tables while preserving anonymity.

Similarly, participating OPOs were assigned anonymous IDs (OPO1-OPO6) that do not reveal the identity of the contributing organization. These identifiers provide a consistent way to stratify analyses by OPO while maintaining confidentiality of the contributing institutions.

We discuss each of the three table categories in further detail below. An additional table, NoteEvents, is expected to be made available in a future release of ORCHID.

### OPO Referrals

The OPO Referrals table contains a row for each of the 133,101 deceased donor referrals (of which 8,972 led to organ donation) across 13 states. We only include data on patients who were mechanically ventilated (a pre-requisite for organ donation) and referred for organ donation (as opposed to tissue or cornea). The table is unique at the patient level (identified by PatientID column) and contains three types of information:

- **Referral information:** Demographics (including age, race, and gender) and description of a patient’s death (cause, mechanism, circumstances, brain death or cardiac death).
- **Outcomes:** Binary indicators for if the patient was approached, authorized, or procured. If procured, the outcome of each organ recovered from the patient (i.e., successfully transplanted, procured but not transplanted, procured for research).
- **Process data:** Timestamps that capture every action taken by OPO staff in relation to a referral, including approach, authorization, and final procurement. The table also includes timestamps for patient brain death (if applicable) and asystole.

The table has 38 columns. Each column is described in detail in an accompanying data dictionary, which includes the variable name, a description of the information recorded, a data type (integer, decimal, string, etc) and notes relevant for reuse. Note that the data for OPOs 1, 2, and 4 does not cover the entire study period. OPO 1 covers Jan 1, 2015 - Nov 23, 2021, OPO 2 covers Jan 1, 2018 - December 31, 2021, and OPO 4 covers Jan 1, 2015 - December 13, 2021. Further, note that the gender and race columns reflect the data stored under these column headers in the underlying electronic health records, and may not reflect or represent the gender, sexual, or racial-ethnic identities of the individuals in the data^16^.

### OPO Events

OPO events are charted across eight tables that span a patient’s hospital stay and the subsequent procurement process. The information in these events tables was entered by OPO staff into the database during the procurement process. A PatientID is used to link the charted events to OPO Referrals. Each charted event is time-stamped (note that the timestamps are date-shifted as described in section 5). The eight events tables are:

- **ChemistryEvents** (ChemistryEvents.csv): Laboratory test results for blood chemistry. Includes several donation-relevant tests including kidney panel (e.g., creatinine), liver function tests (e.g., bilirubin), and electrolyte panel (e.g., sodium, potassium).
- **CBCEvents** (CBCEvents.csv): Complete blood count (with differential) test results.
- **ABGEvents** (ABGEvents.csv): Arterial blood gas (ABG) test results. Includes relevant variables (e.g., partial pressure of oxygen) along with ventilator setting at time of measurement.
- **SerologyEvents** (SerologyEvents.csv): Results of serological testing. Conveys presence of antigens and antibodies that may affect donation (e.g., HIV, hepatitis C, etc.)
- **CultureEvents** (CultureEvents.csv): Results of culture tests for infection. Includes results for blood, urine, and other body fluids.
- **HemoEvents** (HemoEvents.csv): Hemodynamics (e.g., average blood pressure, heart rate) over a range defined by provided timestamps.
- **FluidBalanceEvents** (FluidBalanceEvents.csv): Fluid balance (e.g. average urine output) over a range defined by provided timestamps.
- **NoteEvents** (NoteEvents.csv): Free-text notes made by OPO staff during the process of procurement, documenting processes, outcomes, and events. Due to the challenges of de-identification, the notes are not made available in the current public release of ORCHID, but they are expected in a future release.

### OPO Deaths

The OPO Deaths table reports annual estimates of the number of CALC deaths in each OPO’s service area. We provide a lower bound (+calc_deaths_lb+), upper bound (+calc_deaths_ub+), and median estimate (+calc_deaths+) of the number of CALC deaths in each OPO’s DSA for every year between 2015 and 2021.

## Technical Validation

We collaborated closely with an interdisciplinary team of scientists and organ procurement staffers to evaluate data during development and to conduct quality reviews of data and code.

We intentionally adopted a “minimal preprocessing” philosophy in developing ORCHID. Our goal was to maximize transparency and avoid introducing analytic bias by cleaning or altering data^17^. Preprocessing steps were therefore limited to structural transformations: mapping OPO-specific fields into a harmonized schema, applying date-shifting for HIPAA-compliant de-identification, and generating unique identifiers. Importantly, we did not attempt to correct, impute, or filter values that appeared implausible (e.g., extreme laboratory values, missing demographic fields).

To ensure fidelity, the harmonized data were returned to data analysts at each OPO for review. Each OPO confirmed that the final dataset accurately matched the information in their source donor management systems (iTransplant or TrueNorth).

As part of quality assurance, we performed:

- Schema consistency checks to confirm that required columns were present, datatypes were preserved, and relationships between tables (e.g., PatientID linking across events and referrals) were maintained.
- Chronological checks to confirm that key timestamps were logically ordered (e.g., referral recorded prior to approach).
- Cross-OPO alignment checks to validate that fields with equivalent meaning were mapped consistently across systems.

Beyond these structural and consistency checks, we have deliberately left the raw values intact to support unbiased secondary analyses. Researchers should therefore be aware that the dataset reflects the documentation practices and limitations of the underlying OPO systems.

### Code quality

While creating ORCHID, we endeavored to follow best practice in scientific computing. We used version control (git) to track code contributions, maintained a ticket system to raise and resolve issues, and developed a testing framework to promote software integrity. The process for transforming OPO data to the final ORCHID dataset is reproducible, simplifying the creation of new data releases.

### Patient Attrition

**Fig. 2:**
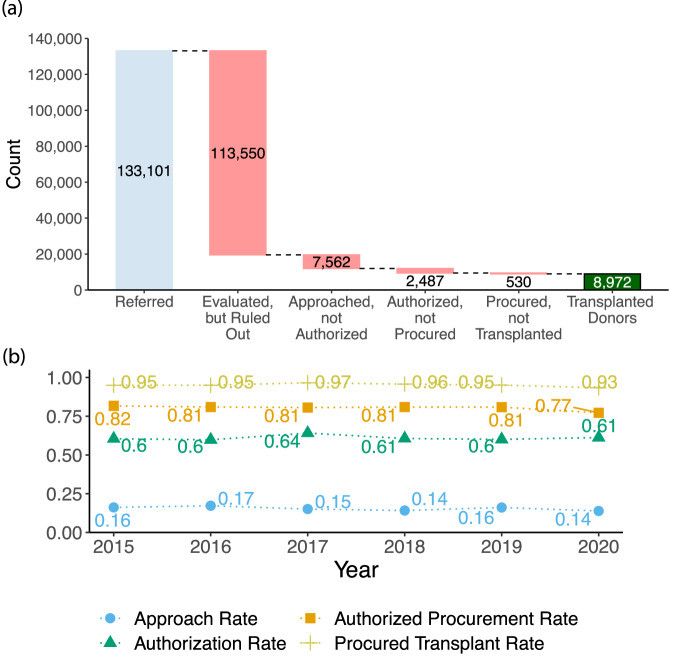
Patient attrition during the organ procurement process. (**a**) Patient attrition during the organ procurement process for all OPOs in our ORCHID. Only 7% of referrals (8,972 out of 133,101) result in successful organ donations. (**b**) Variation of OPO process metrics over time.

In Fig. 2a we show the outcomes of all patients referred for organ donation to the six OPOs in ORCHID. The vast majority of referrals (85%) were ruled out during evaluation. A further 8% of referrals were lost during the approach, authorization, and procurement steps. Ultimately, only 7% of referrals resulted in a successful donation.

Based on discussion with staff of the OPOs, we defined four process metrics that remained broadly consistent over the data collection period:

- **Approach Rate:** Proportion of referred patients whose next-of-kin were approached.
- **Authorization Rate:** Proportion of approached patients who were authorized for donation.
- **Authorized Procurement Rate:** Proportion of authorized patients who provided one or more organs.
- **Procured Transplant Rate:** Proportion of procured patients who led to successful transplants.

We confirmed that these metrics remained similar over the ORCHID data (see Fig. 2b). As expected, there is minor variation between years, which may provide insights upon deeper analysis.

### Linkage to External Data Sources

CALC deaths allow researchers to compute measures of OPO performance that are consistent with federal regulation. As part of the new Final Rule^11^, CMS will assess OPO performance using Donation Rate, defined as the number of donors divided by the number of CALC deaths in the OPO’s service area. CMS defines a donor as a deceased individual from whom at least one organ is transplanted or whose pancreas is recovered for research^11^.

**Fig. 3:**
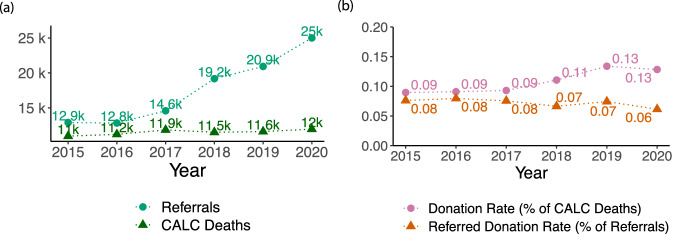
Value of CALC deaths (from external data). (**a**) Comparison of CALC deaths (from external data) in the service areas of the six OPOs and total number of referrals received by the OPOs (from internal OPO data). (**b**) Comparison of the Referred Donation Rate (uses only internal OPO data) and the Donation Rate (uses CALC deaths). As shown in a), referrals as measured in ORCHID data (i.e. from expired patients) greatly outpace CALC deaths in more recent years. In b), we compare the corresponding measures of OPO performance: Donation Rate (uses CALC deaths as a denominator) and Referred Donation Rate (uses referrals as a denominator).

An alternate metric of performance that relies only on OPO internal data is the Referred Donation Rate, defined as the number of donors divided by the number of referrals received by the OPO. Figure 3 illustrates the inconsistency of this internal OPO metric compared to CMS’ regulatory standard. While the number of referrals received by the six OPOs in ORCHID greatly increased between 2015 and 2020, the number of CALC deaths remained flat (Fig. 3a). Similarly, though the Referred Donation Rate (of all OPOs in aggregate) showed a slight decline in this time period, the Donation Rate increased.

The difference in these metrics is likely driven by known variability in referral practices by hospital, OPO, and year^4,10^. This simple analysis demonstrates that relying solely on OPO internal data can lead to misleading conclusions about OPO performance. We provide CALC deaths as part of ORCHID to help researchers avoid such pitfalls and assess OPO performance in a manner consistent with federal regulation.

## Usage Notes

As stated publicly, the goal of this work is to provide essential insights for ongoing federal policymaking, with the ultimate objective of improving OPO performance and addressing health inequities^8^. ORCHID allows researchers to audit both the efficiency and equity of the current procurement process. The dataset not only conveys donor attrition at each stage of the process, but also the individual characteristics of the potential donors who do not lead to transplants. Using ORCHID with tools from process mining, operations research, and economics can provide insight into process and policy changes to improve OPO performance.

The dataset may also offer opportunities to design computational tools for better organ procurement. The detailed clinical data available in ORCHID enables prediction of outcomes such as transplant, offering the opportunity for early identification of potential donors. These models may form the basis of decision-support tools that may ultimately be deployed in OPOs.

We conclude by emphasizing that organ procurement is an urgent public health issue. Bipartisan Members of Congress have called for OPO process data to be publicly available for evidence of effective and equitable service^18,19^. The first public release of ORCHID represents an important step in this direction: the participants in the project have demonstrated their commitment to transparency and are establishing a path towards a larger, nationally representative dataset.

### Limitations on comparability across centers

While the participating OPOs use similar donor management systems, local configuration and documentation practices vary. We harmonized key concepts (e.g., demographics, referral outcomes, laboratory results) into a common structure to facilitate cross-center analysis. However, measurements and procedures are not guaranteed to be fully standardized across sites, and practices may also evolve over time (e.g., if a laboratory test was introduced, retired, or renamed during the study period). These differences were not retrospectively reconciled in ORCHID. Researchers should therefore exercise caution when comparing values across centers or across years, and should consider center-specific practices when interpreting results.

## Data Availability

ORCHID v2.1.1 is available on PhysioNet^15^. To access the data, users must: • Provide profile information, including name and organization, to establish a known identity. • Sign a Data Use Agreement, stating - among other things - that data will not be shared, nor will there be attempts to re-identify patients. Once approved for access, users may download data directly from the ORCHID project on PhysioNet. The data is also made available on Google BigQuery, a cloud database engine, eliminating the need for download and facilitating exploration and analysis.

## Code availability

We have created a publicly available repository of code to support collaborators working on ORCHID, which is available on GitHub: https://github.com/MLforHealth/ORCHID. All code in the repository is made available under the permissive MIT license, enabling broad reuse. We have added sample code for exploring and analyzing ORCHID to the repository, including code to reproduce the figures and tables reported in this paper. We are committed to working with the community to expand and enhance the repository over time.
